# Connective tissue graft with injectable platelet rich fibrin for treatment of RT1 gingival recession by tunnel technique: a randomized clinical and immunohistochemical study

**DOI:** 10.3389/froh.2026.1801423

**Published:** 2026-06-10

**Authors:** Mohammed Nasser Abdel-aziz Mahmoud, Abdelnasser Mohammed Hashem Elrefaei, Mona Saad Shata, Dinesh Rokaya, Shaimaa Mohammed Morsy

**Affiliations:** 1 Department of Oral Medicine & Periodontology, Faculty of Dentistry, Suez Canal University, Ismailia, Egypt; 2 Department of Oral Pathology, Faculty of Dentistry, Suez Canal University, Ismailia, Egypt; 3 Clinical Sciences Department, College of Dentistry, Ajman University, Ajman, United Arab Emirates; 4 Center of Medical and Bio-Allied Health Sciences Research, Ajman University, Ajman, United Arab Emirates

**Keywords:** gingival recession, injectable platelet-rich fibrin, minimally invasive periodontology, periodontal plastic surgery, tunnel technique

## Abstract

**Objectives:**

The literature regarding the use of injectable platelet- rich fibrin (i-PRF) for root coverage is scarce. Therefore, this RCT aimed to evaluate and compare the effect of using connective tissue graft (CTG) with and without i-PRF using the tunnel technique for Cairo class 1 gingival recession (RT1) clinically and immunohistochemically.

**Methods:**

Thirty patients were randomly allocated into 2 groups. (a) CTG immersed in i-PRF (treatment group) and (b) CTG only (control group). The clinical outcomes were assessed at baseline and 6 months. Primary outcome measures were keratinized tissue height (KTH), recession depth (RD), mean root coverage (MRC) & complete root coverage (CRC). Secondary outcome measures were clinical attachment level (CAL), recession width (RW), gingival thickness (GT), probing depth (PD), and Visual analogue scale (VAS) for pain. The Immunohistochemical analysis of vascular endothelial growth factor A (VEGF A) and transforming growth factor beta 1 (TGFβ1) in CTG was done.

**Results:**

The primary outcomes analysis showed statistically significant improvements in KTH and RD at 6 months compared to baseline in both groups (*p* < 0.05). The test group showed significant improvement compared to the control group in 6 months follow up period regarding increased KTH, decreased RD, and MRC. The CRC showed no significant difference between the two groups. The secondary outcomes analysis showed statistically significant improvements in RW, GT, PD, and CAL in 6 months compared to baseline within each group (*p* < 0.05) with no difference between groups. VAS in the test group showed statistically significant improvement compared to the control group at one-week post-operative (*p* < 0.001). The immunohistochemical analysis showed that the CTG of the test group had significantly higher expression of VEGF A and TGFβ1 compared to the control group (*p* < 0.001).

**Conclusion:**

The use of CTG immersed in i-PRF with the tunnel technique for surgical management of RT1 multiple adjacent gingival recessions led to improved KTH, better RD reduction, better MRC and less post-operative pain. Additionally, CTG immersed in i-PRF showed higher expressions of VEGF A and TGFβ1 compared to CTG alone.

**Clinical Trial Registration:** ClinicalTrials.gov, registration NCT06646432.

## 1 Introduction

Gingival esthetic is important for the dentofacial and teeth esthetics (1). Gingival recession (GR) is a periodontal problem, manifested clinically as apical migration of marginal gingiva beyond the cementoenamel junction (CEJ), which leads to root exposure (2). Over 78% of the global population is affected by GR to some extent. It is more prevalent in males and increases with age. Typically, the buccal surfaces are more commonly affected, highlighting the need for interventions to address patients' aesthetic concerns (3). The etiology of GR is multifactorial and can be attributed to many causes, including plaque and calculus deposits (4), aberrant frenal attachments and/ or shallow vestibule (5), faulty tooth brushing (6), orthodontic tooth movement (7), and improper partial denture clasps (8).

There are various systems for GR classification. While Miller's classification (9) was the most widely used system for categorizing GR, other classification systems emerged to overcome its limitations. Cairo classification (10) was proposed in 2011 and has become increasingly used nowadays. It is based on the interdental clinical attachment level in relation to the facial or buccal clinical attachment level. GR is classified into 3 types as follows:

- Recession type 1 (RT1): No interproximal clinical attachment loss (CAL) and CEJ not exposed proximally.
- Recession type 2 (RT2): interproximal CAL same or less than buccal CAL.
- Recession type 3 (RT3): interproximal CAL more than buccal CAL.

It is suggested that the patient may undergo periodontal debridement and monitoring without surgical intervention first if they have a recession depth of 2 mm or less, not undergoing orthodontic or restorative treatment, and clinical attachment loss less than 5 mm (11).

A combination of coronally advanced flap (CAF), combined with connective tissue graft (CTG), is considered the gold standard for surgical management of GR (12). Despite the clinical success and predictability of this technique, it usually encompasses horizontal or oblique papillary incisions and may use vertical releasing incisions in some clinical scenarios. The tunnel technique (TUN) was introduced as a method to access the defect sites without papillary or vertical incisions. This technique has undergone various improvements, starting from creating just a pouch to insert the CTG within (13), to creating a whole tunnel preparation with specialized instruments, inserting the CTG inside, and advancing it coronally (14).

One important advantage of TUN over other techniques is the improved blood supply to the CTG. By eliminating the need for vertical and horizontal incisions, the lateral and papillary blood supply to the CTG is maximized. Using microsurgical tunneling knives during TUN preparation causes less trauma to the gingival tissues, leading to better vascularization of the CTG and reduced risk of its necrosis (14).

Platelet-rich fibrin (PRF) is a second-generation platelet concentrate that includes a high amount of growth factors, platelet-derived growth factor (PDGF), transforming growth factor beta1 (TGF-β1), vascular endothelial growth factor (VEGF), and fibroblast-like growth factor (FGF) (15). Injectable PRF (i-PRF) was first obtained by using low speed centrifugation concept. It was found to contain high amounts of various growth factors, specifically VEGF, TGF β1 & PDGF. i-PRF fabricates a 3D fibrin network trapping platelets, leukocytes, and various growth factors that are released during the process of wound healing, and not only provides us with growth factors but also enhances the handling of grafts and biomaterials used in various periodontal surgeries as well (16). The use of PRF in the surgical management of GR showed improved outcomes in root coverage procedures only in the presence of sufficient gingival thickness (GT) and adequate keratinized tissue height (KTH), making its use alone as a soft tissue graft substitute in root coverage procedures limited, as deficient KTH, GT or both are common in sites with GR (17).

While the use of CTG is known to achieve better clinical results in the treatment of GR, heterogeneous outcomes were reported. The use of materials that are able to stimulate tissue regeneration was introduced to achieve better outcomes (18). Our study was done to clinically evaluate the combination of CTG and i-PRF and use them for GR treatment by TUN. Separately, we evaluated the expression of VEGF and TGF β1 within CTG in both groups.

## 2 Materials and methods

### 2.1 Patients selection and allocation

**Figure 1:**
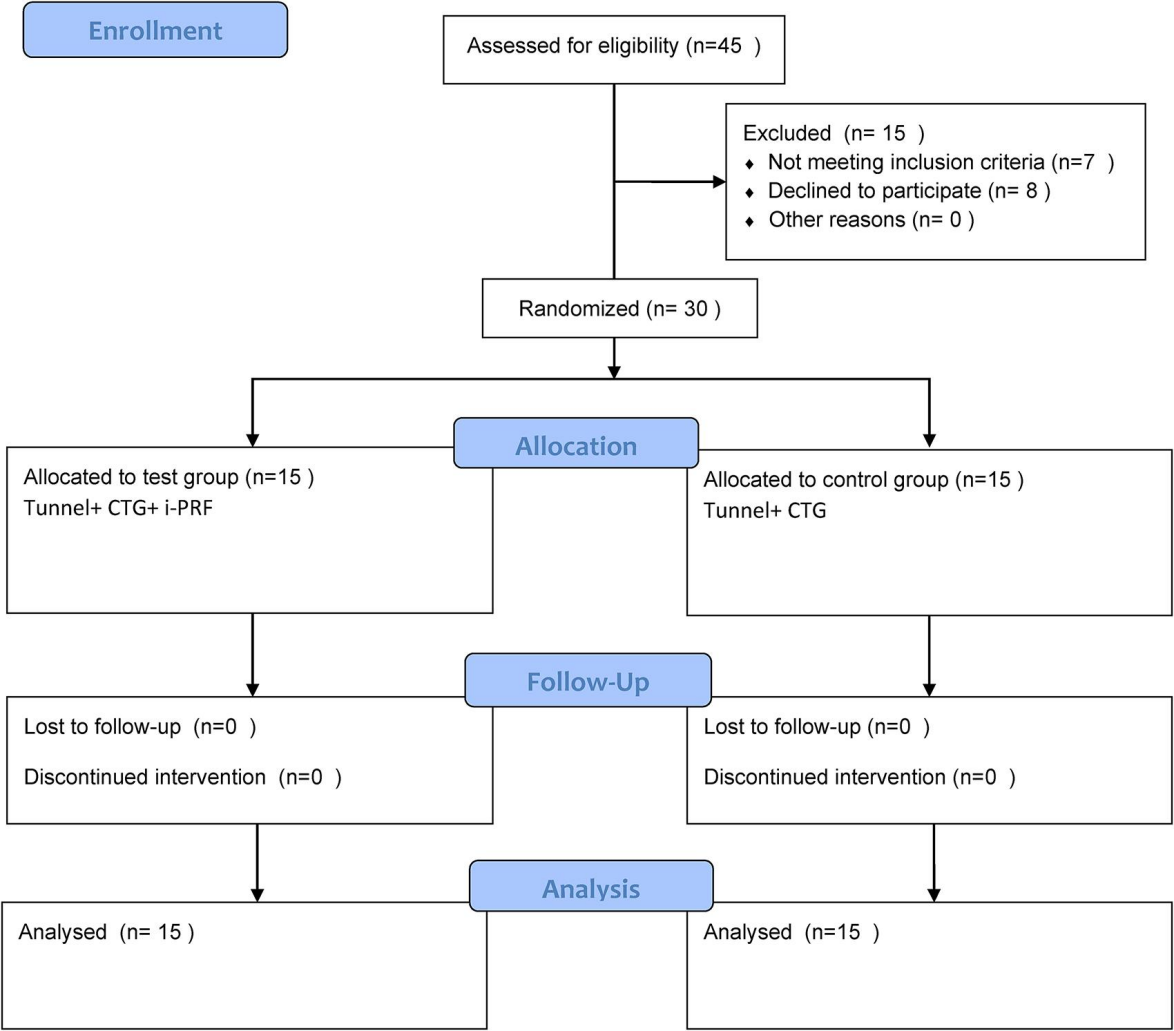
CONSORT flow chart of the patients.

The current study was approved by the ethical committee at the Faculty of Dentistry, Suez Canal University (Approval number: 486/2022) and conducted in accordance with the Helsinki Declaration. Written informed consent was obtained from all participants. All participants were aware of the methods, objectives of the procedures, and the potential complications before signing the informed consent. The study was registered at ClinicalTrials.gov (Registration number: NCT06646432). Patient recruitment was done following the CONSORT flow chart (Figure 1). The participants attending the Periodontology Clinic at the Faculty of Dentistry, Suez Canal University, Egypt, were randomly allocated in this clinical trial with GR based on the following eligibility criteria (19).

Inclusion criteria:

- More than 21 years old
- Systemically healthy
- Presence of RT 1 GR defects
- Presence of identifiable CEJ
- Plaque index score <1.0, indicating low plaque accumulation (20)
- Gingival index score <1.0, indicating very mild gingival inflammation (21)
- Bleeding on probing <10% of all sites (22)

Exclusion criteria:

- Cairo RT2 and RT3 gingival recessions
- Smoking
- Systemic disease that contraindicates surgical procedures.
- Poor compliance with oral hygiene measures
- Necrotizing gingival conditions
- Ongoing active periodontitis

Sample size was calculated using G power statistical power analysis program (version 3.1.9.4) for sample size calculation, A total sample size *n* = 30 (15 in each group) will be sufficient to detect an effect size 1.24 on keratinized tissue height, with an actual power (1-ß error) of 0.95 (95%) and a significance level (*α* error) 0.05 (5%) based on a previous study (23). The result of the sample size calculation revealed that 15 patients were required in each group, with a total of 30 patients for the study. The recruitment date of the patients in this clinical trial was Nov 2024 to March 2025.

### 2.2 Randomization and blinding

Patients were randomly assigned to either the test group (*n* = 15) or the control group (*n* = 15) using a computer-generated randomization sequence. The allocation sequence was concealed using sealed, opaque envelopes. Each envelope was numbered and contained the group assignment for one patient. The envelope was only opened by the surgeon just before the surgical procedure, while treatment for each patient was disclosed only at the time of surgery. One surgeon (MN) performed all surgical procedures to keep the surgical procedures standardized. However, all clinical outcome measurements were performed by a calibrated examiner (M.A) who was blinded to group allocation. Standardized surgical and postoperative protocols were applied to all participants to minimize potential performance bias.

### 2.3 Study outcomes

The clinical outcomes of this trial were recorded at baseline and follow-up periods by the same single-blinded outcome assessor (M.A.) who was blinded to the study details. The primary outcomes were keratinized tissue height, recession depth, mean root coverage, and complete root coverage. The secondary outcomes were postoperative pain, recession width, clinical attachment level, gingival thickness, probing depth, and measurement of vascular endothelial growth factor A and transforming growth factor beta 1 within the graft.

### 2.4 Clinical measurements

Each patient contributed two gingival recession sites (teeth), resulting in a total of 30 sites per group. All treated sites were included in the analysis. Clinical measurements were recorded at the site level for each treated tooth at baseline and at 6 months follow-up. To account for intra-patient variability and avoid the potential violation of independence, site-specific measurements were averaged for each patient at each time point separately (baseline and 6 months), and these mean values were used as the unit of analysis for all statistical comparisons.

The following clinical measurements were recorded:

- Visual analogue scale (VAS) of pain reported by the patient at 7 days after the surgery (24).
- Keratinized tissue height (KTH): the distance from the gingival margin at its deepest recession point to the mucogingival junction (MGJ), which was identified using the roll technique (25).
- Recession depth (RD): the distance from the CEJ to the most apical extension of the gingival margin (25).
- Recession width (RW): the distance from the base of the mesial to the distal papillae along the CEJ (25).
- Gingival thickness (GT): measured by placing an endodontic file with a silicone stopper 2 mm apical to the gingival margin at the middle of the long axis of the tooth (25). The depth of penetration was measured using a ruler (26).
- Probing depth (PD): distance from the gingival crevice to the bottom of the gingival sulcus (27).
- Clinical attachment level (CAL): distance from CEJ to the bottom of the gingival sulcus (27).

These parameters were assessed at baseline and 6 months follow-up.

- Complete root coverage (CRC): the percentage of cases exhibiting a gingival margin that was either level with or positioned coronally to the cementoenamel junction (CEJ) in each group at 6 months follow- up (24).
- Mean root coverage (MRC) at 6 months follow- up: calculated as follows (28)
  $$\begin{array}{rl} & \mathrm{preoperative}\;\mathrm{vertical}\;\mathrm{recession}-\mathrm{Postoperative}\;\mathrm{vertical}\;\\ & \;\mathrm{recession}/\mathrm{preoperative}\;\mathrm{vertical}\;\mathrm{recession}\times 100\text{\%}. \end{array}$$

A UNC 15 probe was used for recording various clinical measurements. All measurements were made in mm.

### 2.5 Pre-operative care

All participants received professional mechanical plaque removal (PMPR). They were encouraged to take oral hygiene measures and advised to use the modified bass technique for toothbrushing. A follow-up after one month was done to reassess the need for surgery and to evaluate the achievement of proper oral hygiene (29). Patients who were eligible to surgical intervention were informed about the details of the procedure and signed an informed consent.

### 2.6 Preparation of i-PRF

10 mL of intravenous blood was collected in tubes without anticoagulant and centrifuged immediately at 700 RPM for 3 min at 60 g at room temperature. The upper liquid layer was then collected as i-PRF (30).

### 2.7 Tunneling surgical technique

After using a local anesthetic containing 4% articaine and 1:100,000 epinephrine (Alexandria Co. for Pharmaceuticals, Alexandria, Egypt), the surgical procedure started by performing root planning on exposed root surfaces using Gracey curettes. Then, TUN preparation was made through the buccal gingivae using microsurgical tunneling knives (Sedra Dent Co., Cairo, Egypt) that were advanced slowly and gently and extended beyond MGJ to allow mobility of the tissues coronally without tension. The pouch preparations were connected between adjacent teeth. Inter-dental papillae were detached with full- thickness preparation, creating a tunnel between the adjacent teeth. Any remaining muscle fibers on the inner aspect of the flap alveolar mucosa were cut using Gracey curettes with care to obtain a passive coronal positioning of the flap and the papilla (14, 31).

CTG was harvested from the hard palate as a free de-epithelialized graft with two horizontal and two vertical incisions delineating the graft. At first, the blade was oriented perpendicular to the palate, and once an adequate soft tissue thickness was obtained, it was rotated to be almost parallel to the superficial surface. Upon harvesting, the yellow fatty tissue was eliminated. De-epithelization was done extra-orally using a no.15 blade while keeping the blade parallel to the external surface, and graft thickness was adjusted at 1 mm uniform thickness. The different consistency and light reflection helped ensure clinical removal of epithelium (24).

**Figure 2:**
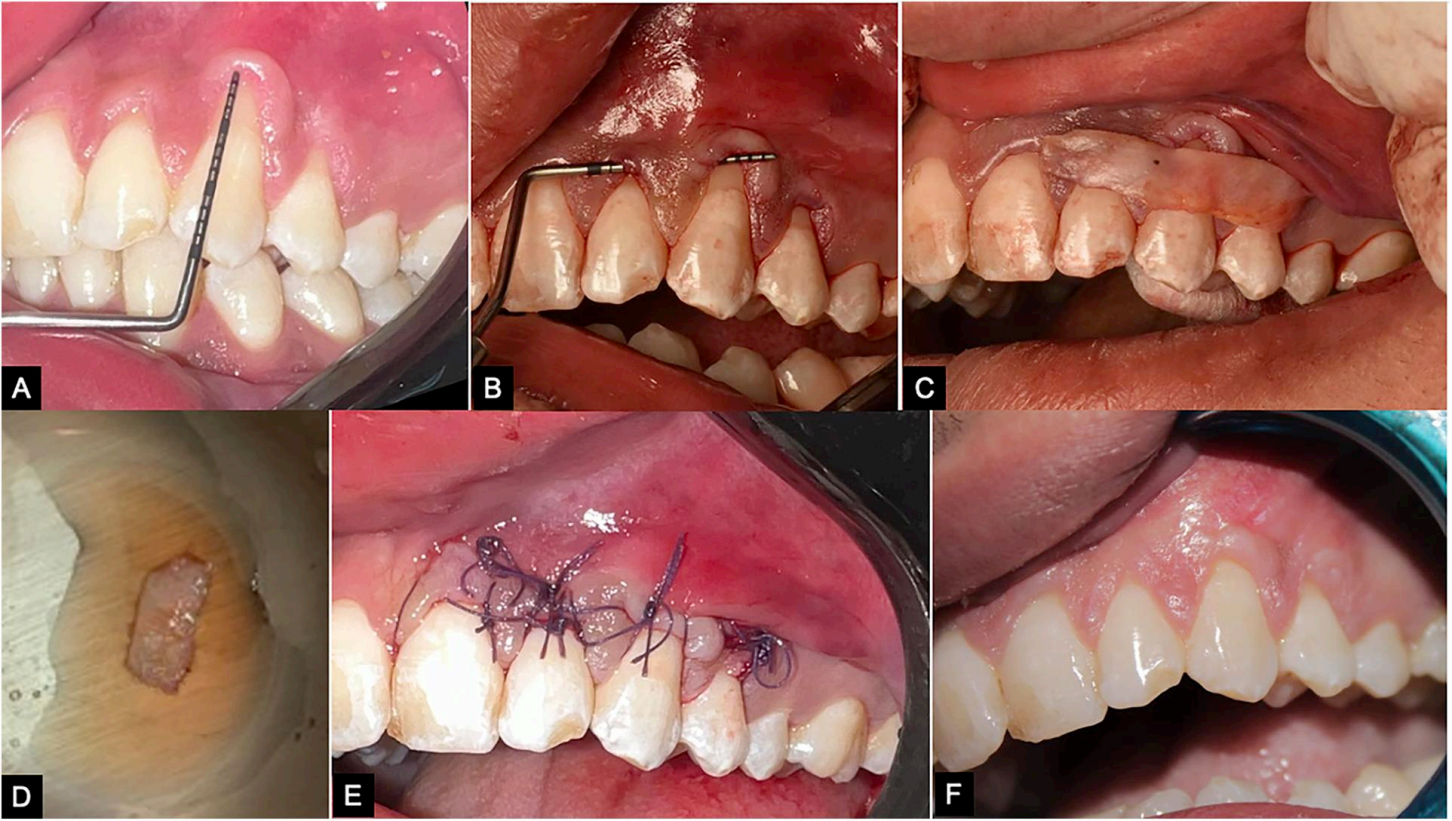
Tunneling procedure in the test group. **(A)** Initial situation showing recession at the upper lateral incisor, canine, and 1st premolar. **(B)** Tunneling. **(C)** CTG. **(D)** CTG immersed in i-PRF. **(E)** Suturing. **(F)** 6 months follow-up.

**Figure 3:**
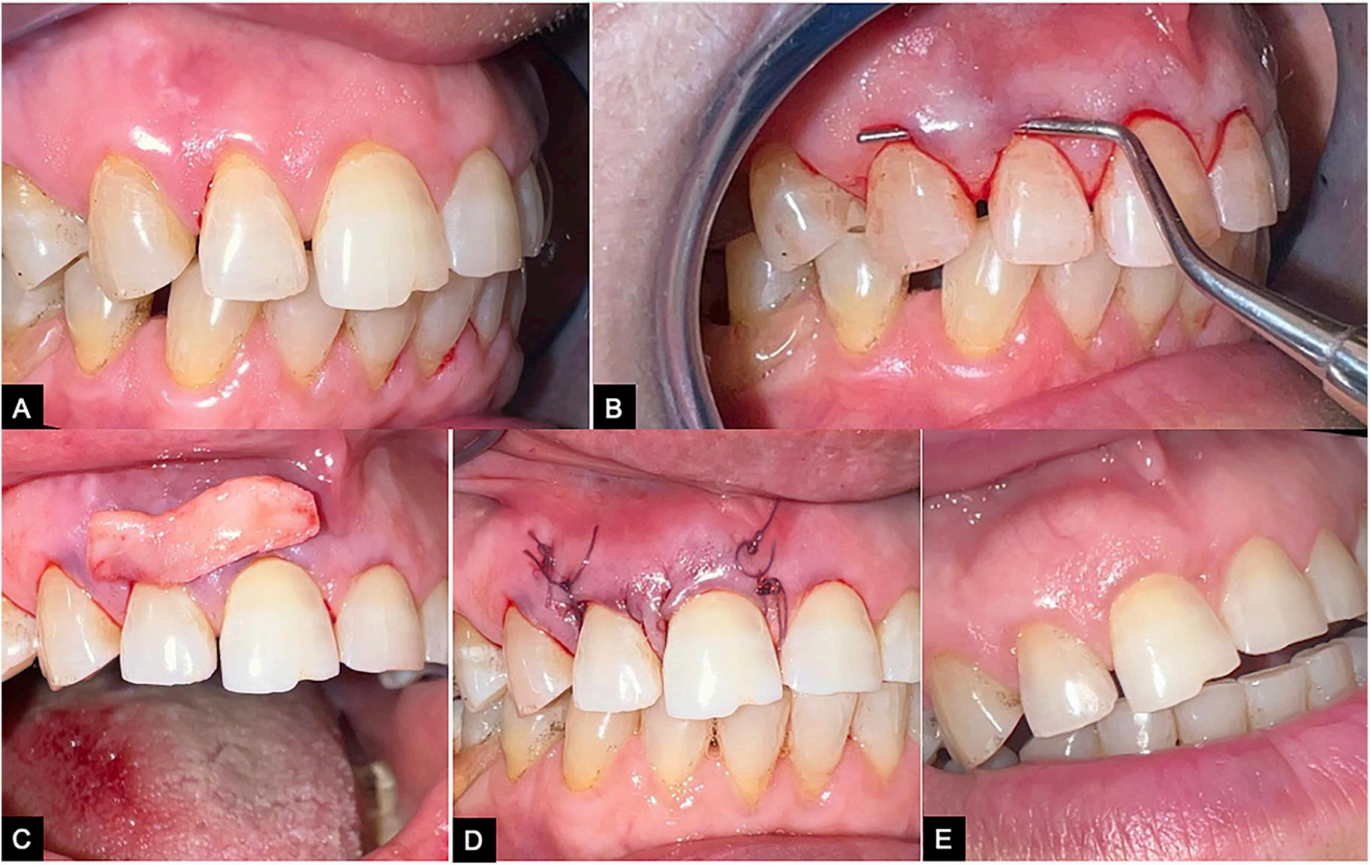
Tunneling procedure in the control group. **(A)** Initial situation showing recession at the upper central and lateral incisors. **(B)** Tunneling with a periodontal probe passing through the prepared tunnel. **(C)** CTG. **(D)** Suturing. **(E)** 6 months follow-up.

In the test group only, CTG was immersed in i-PRF for 15 min (32). A section of CTG was cut from the graft and stored in a jar containing 10% neutral buffered formalin for immunohistochemical staining. The exposed root surfaces were instrumented using Gracey curettes (Sedra Dent Co., Cairo, Egypt). The CTG was slid through the prepared tunnel by suture and pushed inside the pouch by the packing instrument. Then, the gingivopapillary complex was coronally advanced using a vertical mattress suture at each papilla. In the control group, the same procedures were done without the use of i-PRF (14). Figure 2 demonstrates the steps of the procedure made in the test group during the surgical intervention till the 6-month follow-up period, while Figure 3 demonstrates the clinical steps in the control group.

### 2.8 Post-operative care

The patients were instructed to use ibuprofen 600 mg tablets for pain and to reduce possible edema resulting from surgical procedures. The patients were advised not to use tooth brushing at the surgical site and to use only 0.2% chlorohexidine digluconate mouthwash for 2 weeks (14).

### 2.9 Immunohistochemical analysis

The graft samples were fixed in 10% formalin for 24–48 h at room temperature. The tissues were then dehydrated through a graded series of ethanol, cleared in xylene, and infiltrated and embedded in paraffin wax using a standard tissue processor. Serial sections were cut at a thickness of 4–5 µm using a rotary microtome. The sections were mounted on positively charged glass slides to ensure adhesion during subsequent procedures.

The immunohistochemical localization of VEGF-A (Gene Tex®, USA) and TGF-β1 (Abclonal®, USA) was achieved following a standardized protocol. Deparaffinization and heat-induced antigen retrieval, endogenous peroxidases, and non-specific binding sites were blocked. Sections were then incubated with specific primary antibodies against VEGF-A and TGF-β1, followed by appropriate secondary antibodies and a streptavidin-HRP complex. The reaction was visualized using a DAB chromogen, resulting in a brown precipitate at the antigen site, and tissues were counterstained with hematoxylin to provide morphological context.

The immunohistochemical investigation of the tissue was carried out by two independent pathologists. The images were captured by an E-330 Olympus digital camera at 400× objective magnification. Quantitative analysis of immunohistochemical staining for VEGF-A and TGF-β1 was performed using ImageJ software (ImageJ/Fiji 1.46). Digitized images were processed using color deconvolution. A consistent threshold was applied to identify positively stained areas within defined regions of interest. The key output metrics were the Integrated Density (the sum of pixel values) and the Area (in pixels) of the stained regions. The quantitative value for protein expression was calculated as Mean Intensity (Integrated Density/Area) for each field. A minimum of five fields per tissue section were analyzed, and the results for each sample were averaged (33, 34). The immunohistochemical analysis was done in the Arbitrary Unit (AU).

### 2.10 Statistical analysis

Statistical analysis was performed using SPSS software for Windows (version 22.0; IBM Corp., Armonk, NY, USA), with the level of significance set at *P* < 0.05. As each patient presented with multiple treated sites, clinical data were initially collected at the site level and subsequently aggregated at the patient level by calculating the mean of site-specific measurements for each patient at each time point. Accordingly, the patient was considered the statistical unit of analysis to avoid clustering effects and violation of independence.

Following aggregation, data distribution was assessed for normality using the Shapiro–Wilk test, and the choice of statistical tests and data presentation was based on the distribution of each variable. Age, RD, CAL, and growth factor data showed normal distribution, while the remaining parameters were non-normally distributed. Variables with normal distribution were expressed as mean ± standard deviation (SD) and analyzed using parametric tests (paired t-test for within-group comparisons and independent t-test for between-group comparisons). Variables with non-normal distribution were expressed as median and range and analyzed using non-parametric tests (Wilcoxon signed-rank test for within-group comparisons and Mann–Whitney U test for between-group comparisons). Qualitative data were presented as frequencies and percentages, and comparisons between groups were performed using the Chi-square test.

## 3 Results

### 3.1 Baseline characteristics

This study was carried out to evaluate the effect of CTG immersed in i-PRF for the surgical management of GR using TUN. A total of 45 patients from the periodontology clinic at the Faculty of Dentistry, Suez Canal University were assessed for appropriateness; 7 patients did not meet the eligibility criteria and were excluded, while 8 refused to participate and 30 patients with RT1 GR were recruited and assigned randomly into two groups: CTG + i-PRF (*n* = 15, test group) or CTG alone (*n* = 15, control group). The patients from the CTG + i-PRF group were 8 females and 7 males with a mean age of 39.3 years ± 7.7. In the CTG group, 8 males and 7 females were recruited with a mean age of 37.1 years ± 7.2 years. Regarding the examined teeth, the CTG + i-PRF group included 22 teeth (73.3%) in the maxilla and 8 teeth (26.7%) in the mandible, while the CTG group included 24 teeth (80%) in the maxilla and 6 teeth (20%) in the mandible (Table 1).

**Table 1:** Demographic data and their comparisons.

Parameter	Test Group Mean ± SD	Control Group Mean ± SD	*P*-value
Age (years) (Mean ± SD)	39.3 ± 7.7	37.1 ± 7.2	0.42
Gender (*n*, %)			
Male	7 (46.7%)	8 (53.3%)	1
Female	8 (53.3%)	7 (46.7%)	
Arch (*n*, %) (Anteriors and premolars)	Maxillary: 22 (73.3%)	Maxillary: 24 (80%)	0.7
	Mandibular: 8 (26.7%)	Mandibular: 6 (20%)	

SD, standard deviation.

### 3.2 Clinical parameters

#### 3.2.1 Primary outcome clinical measures

Keratinized tissue height (KTH) measured in (mm): A significant increase was seen in both groups at 6 months compared with the baseline (*p* value <0.001). The test group showed a significantly higher increase in 6 months compared to the control group (*p* value <0.001) with a large effect size 0.85 suggesting a notable difference which may be clinically relevant for increasing keratinized tissue height and improving gingival stability (Tables 2, 7).

**Table 2:** Comparison between non-parametric clinical data (mm).

Parameters	Intervals	Test Group MD (Min-Max)	Control Group MD (Min-Max)	CI 95%		Effect size	*P*-value
				Lower	Upper		
KTH	Baseline	3.50 (2.50–4.00)	3.50 (2.75–4.50)	−0.298-	0.331	0.08	0.68
	6 months	6.00 (5.00–6.75)	5.00 (4.50–6.00)	0.595	1.2713	0.72	<0.001**
RW	Baseline	4.00 (3.00–5.00)	4.00 (3.00–5.00)	−0.397-	0.530	0.003	0.838
	6 months	0.75 (0.00–2.00)	1.00 (0.00–2.00)	−0.746-	0.279	0.03	0.389
GTH	Baseline	1.25 (0.75–1.50)	1.25 (0.75–1.50)	−0.113-	0.179	00	0.71
	6 months	2.00 (1.75–2.25)	2.00 (1.50–2.25)	−0.142-	0.109	0.003	0.653
PD	Baseline	1.25 (0.75–2.00)	1.25 (0.50–2.00)	−0.345-	0.212	0.057	0.728
	6 months	1.00 (0.50–1.50)	1.00 (0.50–1.50)	−0.222-	0.222	0.0	0.902
VAS at 7 days		5.00 (4.00–7.00)	7.00 (6.00–8.00)	−2.079–2.0101		0.67	0.001**

MD, median; Min, minimum; Max, maximum; CI, Confidence intervals.

* Statistically significant at *P*-value < 0.05.

** Highly statistically significant *P*-value < 0.001.

Recession depth (RD) measured in (mm): A significant decrease was observed in both groups at 6 months compared with the baseline (*p* value <0.001). The test group showed a significantly higher decrease in 6 months compared to the control group (*p* value <0.001) with a large effect size 0.9 suggesting a notable difference between groups which may be clinically relevant for reducing depth of gingival recession (Tables 3, 6).

**Table 3:** Comparison between parametric clinical data (mm).

Parameters	Intervals	Test Group (Mean ± SD)	Control Group (Mean ± SD)	CI 95%		Effect size	*P*-value
				Lower	Upper		
RD	Baseline	3.43 ± 0.46	2.93 ± 0.80	0.013	0.987	0.136	0.045
	6 months	0.38 ± 0.31	0.78 ± 0.60	−0.756-	−0.044-	0.159	0.029*
CAL	Baseline	4.63 ± 0.50	4.25 ± 0.79	−0.111-	0.878	0.083	0.12
	6 months	1.68 ± 0.43	1.38 ± 0.44	−0.025-	0.625	0.113	0.069

SD, standard deviation; CI, Confidence intervals.

* Statistically significant at *P*-value <0.05.

Mean root coverage (MRC) (%): The test group showed significantly higher values compared to the control group at 6 months (*P*-value <0.05) with a small effect size 0.22 suggesting a small but measurable magnitude of difference which may be clinically relevant for improving the extent of root coverage (Table 4).

**Table 4:** Comparison between groups regarding percentage of MRC and CRC (%).

Parameter	Test group Median (min-Max)	Control group Median (min-Max)	CI 95%		Effect Size	*P*-value
			Lower bound	Upper bound		
MRC (%)	0.90 (0.75–1.00)	0.67 (0.50–1.00)	3.579	24.154	0.22	0.033*
CRC (%)	50%	43.3%				0.39

MD, median; Min, minimum; Max, maximum; CI, Confidence intervals.

* Statistically significant at *P*-value <0.05.

Complete root coverage (CRC) (%): No significant difference between the two groups (Table 4).

#### 3.2.2 Secondary outcome clinical measures

Recession width (RW) measured in (mm): A significant decrease was seen in both groups at 6 months compared with the baseline (*p* value <0.001) without a significant difference between the two groups (Tables 2, 7).

Gingival thickness (GT) measured in (mm): A significant increase was observed in both groups at 6 months compared with the baseline (*p* value <0.001) without a significant difference between the two groups (Tables 2, 7).

Probing depth (PD) measured in (mm): A significant decrease was seen in both groups at 6 months compared with the baseline (*p* value <0.001) without a significant difference between the two groups (Tables 2, 7).

Clinical attachment level (CAL) measured in (mm): A significant increase was seen in both groups at 6 months compared with the baseline (*p* value <0.001) without a significant difference between the two groups (Tables 3, 6).

Visual analogue scale (VAS): The test group showed statistically significantly lower scores of pain compared to the control group after one week of the surgical procedure (*P* < 0.001) with moderate effect size 0.67 suggesting notable difference between both groups which may be clinically relevant for post operative pain experienced by the patient at 7 days (Table 2).

VEGF A & TGF β1 measured in arbitrary units (AU): Both growth factors showed highly significant scores in the CTG immersed in i-PRF analyzed in the CTG + i-PRF group, compared to the CTG alone from the CTG group (*P*-value < 0.001), with large effect sizes for both growth factors suggesting a substantial magnitude of difference in growth factors expression between groups (Table 5).

**Table 5:** Results of immunohistochemical tests in the arbitrary unit (AU).

Parameter	Test Group Mean ± SD	Control Group Mean ± SD	CI 95%		Effect size	Inter group *P*-value
			Lower bound	Upper bound		
VEGF A	180.46 ± 11.24	71.97 ± 8.29	101.106	115.884	10.98	<0.001**
TGFβ1	161.56 ± 5.34	74.24 ± 10.00	81.328	93.317	10.89	<0.001**

SD = standard deviation.

** Highly statistically significant at *p* < 0.001.

**Figure 4:**
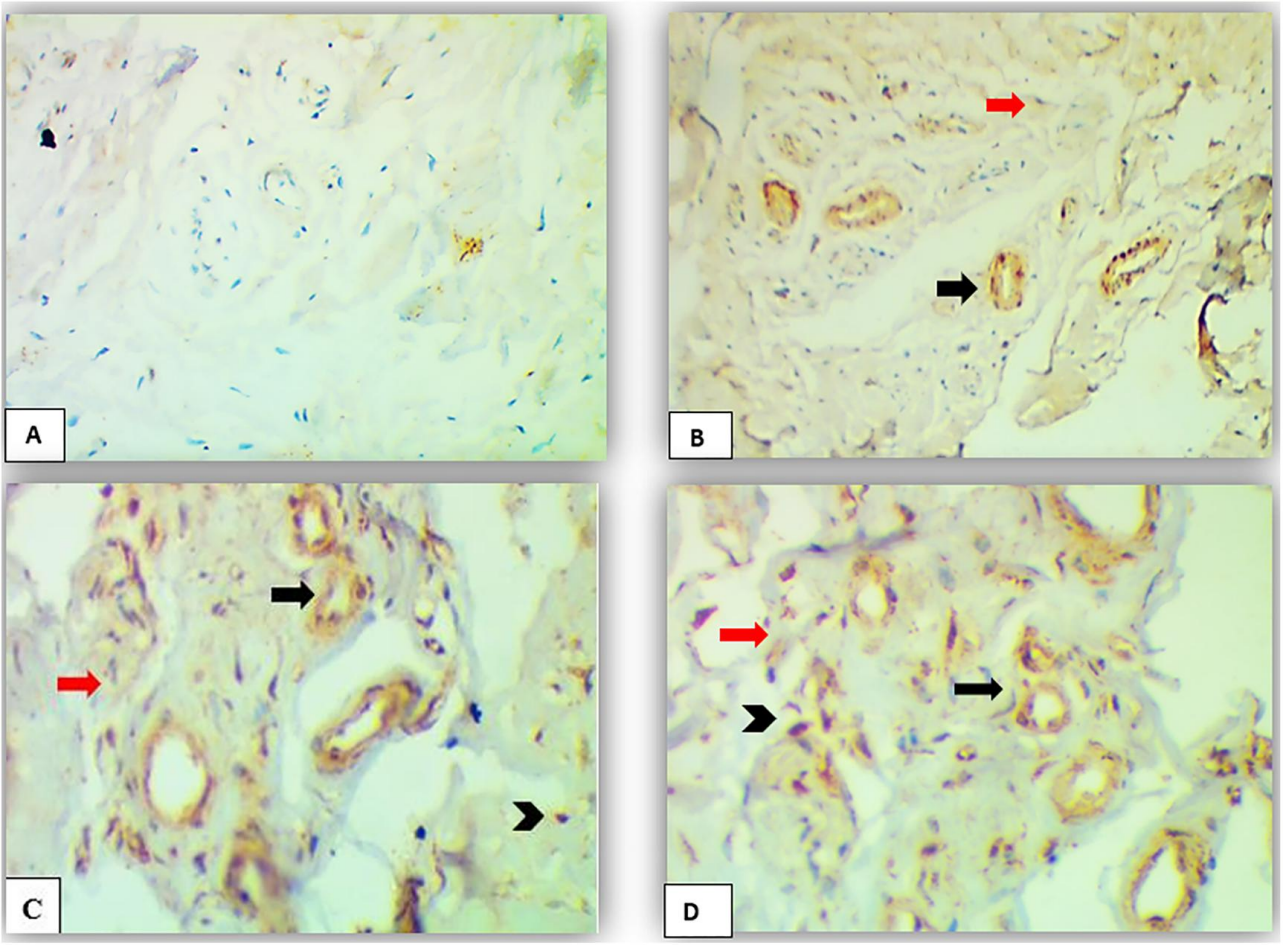
Immunohistochemical expression of VEGF A. **(A)** Mild positive expression in endothelial cells and fibroblasts, with negative expression in chronic inflammatory cells in the control group (ABC-DAB ×20). **(B–D)** Strong positive expression in endothelial cells (black arrow), chronic inflammatory cells (arrowhead), and fibroblasts (red arrow) in the test group (ABC-DAB ×40).

**Figure 5:**
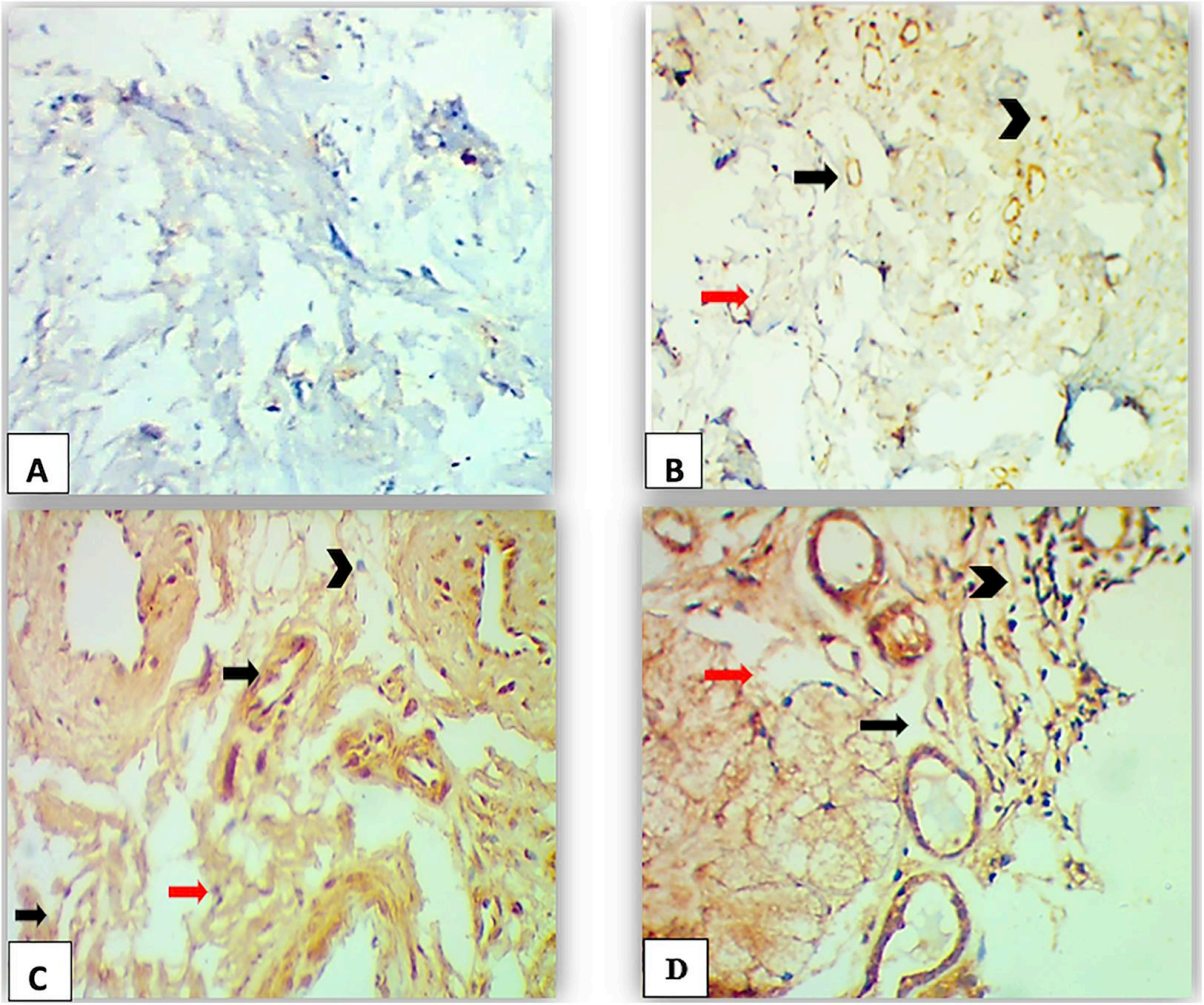
Showing immunohistochemical expression of TGF-β1. **(A)** Mild positive expression in endothelial cells and fibroblasts (ABC-DAB ×20). **(B–D)** Strong positive expression in endothelial cells (black arrow), chronic inflammatory cells (arrowhead), and fibroblast (red arrow) (ABC-DAB ×40).

Figures 4, 5 demonstrate the immunohistochemical expression and staining intensity of VEGF A and TGF-β1 between both groups, respectively. The test group showed higher expression of these growth factors after 15-minute immersion of CTG in i-PRF compared to mild expression in the control group, where no immersion in i-PRF was made. Changes between groups and within the same group were also calculated and presented for parametric (Table 6) and non-parametric (Table 7) clinical data.

**Table 6:** Comparison of changes within and between groups for parametric clinical data.

Parameters	Groups	Mean ± SD	CI 95%		Effect size	*P*-value
			Lower bound	Upper bound		
RD	Test	3.05 ± 0.4246	2.815	3.285	0.9	<0.001**
	Control	2.15 ± 0.4705	1.889	2.411	0.9	<0.001**
	P0	<0.001**				
	Effect size	0.9				
CAL	Test	2.95 ± 0.414	2.721	3.179	0.9	<0.001**
	Control	2.867 ± 0.565	2.553	3.1801	0.9	<0.001**
	Effect size	0.17				
	P0	0.649				

CI, Confidence interval; P0, significance between groups; *P*-value, significance between intervals.

** Highly statistically significant *P* < 0.001.

**Table 7:** Comparison of changes within and between groups for non-parametric clinical data.

Parameters	Groups	MD change (Min-Max)	CI 95%		Effect size	*P*-value
			Lower bound	Upper bound		
KTH	Test	2.5 (2–3.25)	−2.759	−2.375	0.89	<0.001**
	Control	1.5 (1.5–2)	−1.776	−1.524	0.91	<0.001**
	P0	<0.001**				
	Effect size	0.85				
RW	Test	3 (2.5–4)	2.793	3.307	0.88	<0.001**
	Control	3 (2–3.25)	2.561	2.939	0.89	<0.001**
	P0	0.085				
	Effect size	0.314				
GT	Test	0.75 (0.5–1)	−0.907	−0.693	0.89	<0.001**
	Control	0.75 (0.75–1)	−0.920	−0.780	0.91	<0.001**
	P0	0.534				
	Effect size	0.11				
PD	Test	0.25 (0–0.5)	0.169	0.364	0.82	<0.001**
	Control	0.25 (0–0.75)	0.222	0.446	0.84	<0.001**
	P0	0.379				
	Effect size	0.16				

MD, median change; Min, minimum; Max, maximum; CI, Confidence intervals; P0, significance between groups; *P*-value, significance between intervals.

** Highly statistically significant *P* < 0.001.

## 4 Discussion

Platelet concentrates have been used as surgical adjuvants in oral and maxillofacial surgery, orthopedic surgery, and plastic surgery to improve healing and regeneration (35). The main goal of this study was to demonstrate the effectiveness of using i-PRF with CTG together with TUN in RT1 recession cases. A large body of literature supports the use of TUN + CTG for multiple GR defects (36–38). The results of our control group demonstrated clinical outcomes comparable to those reported in the literature (39).

The use of TUN has shown merits supporting its use over other techniques in the management of mucogingival defects. This includes decreased vascular disruption and preservation of interdental papillae by avoiding any vertical or papillary incisions. It provides better blood supply to the graft, achieves better flap stability, and gets rid of potential scar formation (40).

The results of using PRF in the management of GR are diverse between studies, with some studies reporting significant improvement with the use of PRF (41, 42) while other studies found no added benefits (43, 44). The diversity among these results may be explained by the various preparation methods of PRF that lead to different PRF characteristics (45). In addition, we used the liquid form of i-PRF over the conventional PRF membrane to avoid any interruption to the plasmatic circulation of CTG, which is the major source of vascularity to the graft (43).

In our study, there was significant improvement of RD, RW, and CAL in both groups at 6 months follow up compared to baseline. This may be due to the coronal advancement of the gingival margin 1 mm to the CEJ, increasing the possibility of CRC, achieving more RD reduction, MRC, and CAL gain (46). Using CTG is known to stabilize the gingival margin and resist further apical migration. In both groups, GT showed significant increase in the 6 months follow-up compared to baseline. GT increase can be attributed to the use of CTG, which is known to result in thickening of the gingiva that also helps in creeping attachment (47). CTG harvested as FGG and de-epithelialized extra orally, as in our study, is reported to increase GT more than CTG obtained with other techniques, such as the single incision technique, due to its rich content of collagen fibers (48). KTH showed significant increase in the 6 months follow-up compared to baseline in both groups. This can be attributed to the fact that MGJ is genetically predetermined and would regain its original position over time (49). Additionally, it is reported that surface keratinization of the gingival tissue is induced by the genetic information in the palatal CTG (50). The CTG + i-PRF group presented significant improvement compared to the CTG group at 6 months follow-up period in RD reduction, KTH increase, and MRC. VAS score of pain at the early healing period showed a significant difference between both groups in favor of the CTG + i-PRF group. Separately, expression of VEGF A and TGF-β1 in CTG was significantly higher in the test than the control group using immunohistochemical analysis.

To our knowledge, this is the first study to evaluate the expression of growth factors in CTG using immunohistochemical analysis. To assess VEGF-A and TGF-β1 expression, immunohistochemical staining was performed on CTG sections from the control group and on CTG samples immersed in i-PRF in the test group. The test group demonstrated higher expression for VEGF-A and TGF-β1 compared to the control group (Figures 4, 5). It is important to note that immunohistochemical analysis reflects protein expression within tissue sections and does not provide evidence of functional integration or biological incorporation of these growth factors into the graft. VEGF is known to be associated with angiogenesis through stimulation of capillary network formation and neovascularization (51). Similarly, TGF-β1 is associated with the regulation of cell proliferation, differentiation, and migration, and may contribute to fibroblast activity and extracellular matrix production, including collagen, fibronectin, and proteoglycans (52).

The improvements observed in our study are both statistically and clinically meaningful. Both groups showed significant reductions in RD in 6 months compared to baseline. The test group demonstrated a significantly greater reduction compared to the control group at 6 months with a large effect size. This was further represented by the significantly higher MRC achieved in the test compared to the control group. This may be clinically relevant and associated with obtaining more root coverage.

Regarding KTH, both groups showed significant increase in KTH in 6 months compared to baseline. KTH at 6 months exhibited a significant increase in the test group compared to the control group with a large effect size. This may be clinically relevant as expanding the zone of keratinized tissue would enhance gingival margin stability and reduce the risk of relapse over time and may be valuable in areas where insufficient keratinized mucosa would compromise long-term outcomes.

These findings may be associated with i-PRF and its numerous growth factors, which, during early healing stages, may improve vascularity by recruiting endothelial cells and enhancing fibroblast and epithelial cell proliferation. PRF was shown to correspond with more elongated rete pegs of the epithelium extending into the underlying connective tissue and accelerated formation of junctional epithelium and connective tissue attachment, which may support more mechanical resistance to traumatic agents and maintain initial graft stability in early healing stages, which is essential for initial graft survival and integration at the early healing period (53). These associations could contribute, at least partially, to explaining the improved RD reduction, KTH, and MRC in the test group over the control group.

Similarly, VAS at 7 days demonstrated significant difference between both groups in favor of the test group with a moderate effect size reflecting less postoperative pain, which is an important patient-reported outcome measure. The decrease in VAS scores of the test group compared to the control group at 7 days post-operatively can be associated with the rapid progress of wound healing due to the release of various growth factors. It is also reported that PRF can lead to quick natural resurfacing of bone in the surgical site, covering the exposed nerves and decreasing pain perception. PRF has an antagonizing effect against kinins released from the wound during the inflammatory phase (54). It has also shown anti-bacterial effects that would help in reducing post-operative infections that would result in pain (55).

Compared to our study, Turer et al. (32) used CTG after soaking for 15 min in i-PRF for management of a single GR and compared it to CTG alone using CAF in both groups. All clinical parameters in both groups showed significant improvement in the 6-month follow-up period compared to baseline. The test group showed significant improvement over the control group in VAS for post-operative pain. At 6 months, statistical significance improvement in RD and KTH was found in favor of the test group. However, MRC did not show statistical significance at 6 months between the two groups, in contrast to our results. This may be attributed to the MRC achieved by tunneling in multiple adjacent recession defects, which is around 87% (39), while for CAF, in a single tooth recession is reported to be 97% even without the use of any adjuncts (56).

Patil et al. (57) compared CTG soaked in i-PRF for 15 min vs. CTG alone for isolated mandibular anterior teeth with GR using a gingival pedicle split thickness tunnel. The 4-month follow-up revealed a slight, but statistically insignificant, difference between both groups in favor of the i-PRF group, except for wound healing. The difference between these results and our results may be attributed to the difference in the technique used, as the gingival pedicle tunnel technique in this study allowed more ability to close the GR site due to the use of a laterally closed tunnel with a vertical releasing incision, achieving comparable results in both groups. This technique does not change the location of MGJ. The follow-up in this study was shorter than our study and might contribute to the difference in the results (57).

The results of this study need to be interpreted cautiously. A methodological limitation of the present study is that clinical data were analyzed at the patient level, with site-specific measurements aggregated to obtain a single value per patient. Although this approach was adopted to avoid violation of independence due to multiple treated sites within the same patient, it may have resulted in loss of within-patient variability, as differences between individual sites could not be fully captured. In addition, aggregation reduced the effective sample size, which may have had a potential impact on statistical power. Furthermore, this approach limits the interpretation of site-level effects, as outcomes reflect averaged patient responses rather than individual site-specific outcomes. Also, the 6-month follow-up period may be insufficient for creeping attachment to completely occur and to evaluate long-term graft stability and integration. Also, more accurate results may have been obtained with a split-mouth design. In addition, the absence of aesthetic clinical evaluation is considered a major limitation in this study. Regarding sample size calculation, it could be argued that MRC would have been better to be used in the sample size estimation. Our hypothesis was based on the role of the enhanced keratinized tissue height, which would help achieve better recession coverage outcomes and could indirectly affect the gingival margin stability in the long term. Moreover, we acknowledge that no correlation analysis was made between immunohistochemical and clinical results. This would have given more strength to the clinical effects of increased growth factor expression evaluated in this study, rather than being speculative. In addition, our study was not powered to evaluate the depth of penetration of growth factors within the CTG. The immunohistochemical analysis used in our study demonstrated only the localization of growth factors but could not confirm true biological incorporation. Further studies with a focused concern on studying the kinetics of growth factors in respect to the graft are recommended. Also, the earlier timing of evaluating post-operative pain (Day 1–3) would provide a stronger interpretation of the analgesic effect of i-PRF. Finally, the study was single-blinded, which carried the risks of bias from the operator. To minimize bias risks, standardized protocols and objective measurements were performed. The outcome assessor was blinded and not involved in the study.

## 5 Conclusion

From the current study, we concluded that:

1. Surgical management of multiple RT-1 gingival recession defects with the tunnel technique and CTG is an effective treatment modality.
2. The use of CTG immersed in i-PRF for 15 min with the tunnel technique had significantly improved RD reduction, KTH & MRC at 6 months follow-up and decreased post-operative pain assessed by VAS at one week after the surgical procedure when compared to using CTG alone with the tunnel technique.
3. Immersion of CTG in i-PRF for 15 min may be associated with higher expression of VEGF A and TGF β1 compared to CTG alone as assessed by immunohistochemistry.

## 6 Clinical relevance

There is no study comparing the use of CTG immersed in i-PRF for the treatment of multiple adjacent gingival recession defects using the tunnel technique to CTG alone with the tunnel technique. Principal findings: This study showed that i-PRF + CTG + TUN for the treatment of RT1 recession defects may be associated with additional benefits with respect to improved KTH, decreased RD, and better MRC. This study demonstrated the success of TUN + CTG + i-PRF for the surgical management of RT1 multiple adjacent gingival recession sites with better outcomes regarding increased KTH, reduced RD, and increased MRC.

## Data Availability

The datasets presented in this article are not readily available because the data presented in this study are available on request from the corresponding author. Requests to access the datasets should be directed to Dinesh Rokaya, dineshrokaya115@hotmail.com.
